# Empirical evaluation of an ICF core set for managing musculoskeletal conditions in primary care physical therapy services in Spain: a multicenter study

**DOI:** 10.3389/fresc.2026.1960608

**Published:** 2026-09-22

**Authors:** Héctor Hernández-Lázaro, Sandra Jiménez-del-Barrio, María Teresa Mingo-Gómez, Ana Isabel Rodríguez-Fernández, Agnieszka Wiśniowska-Szurlej, Agnieszka Sozańska, Zofia Dzięcioł-Anikiej, Luis Ceballos-Laita

**Affiliations:** 1 Active Coping Strategies for Pain Unit in Primary Care, Soria Healthcare Management, Regional Health Service of Castilla y León (SACYL), Soria, Spain; 2 Faculty of Health Sciences, University of Valladolid, Soria, Spain; 3 Clinical Research in Health Sciences Group, University of Valladolid, Soria, Spain; 4 Cervera de Pisuerga Primary Care Physical Therapy, Palencia Healthcare Management, Regional Health Service of Castilla y León (SACYL), Palencia, Cervera de Pisuerga, Spain; 5 Faculty of Health Sciences and Psychology, Collegium Medicum, University of Rzeszów, Rzeszów, Poland; 6 Department of Personalized Musculoskeletal Physiotherapy, Medical University of Białystok, Białystok, Poland; 7 Physical Activity Research Laboratory, Clinical Research Center, Medical University of Białystok, Białystok, Poland

**Keywords:** international classification of functioning, disability and health (ICF), musculoskeletal diseases, patient outcome assessment, physical therapy modalities, primary health care

## Abstract

**Background:**

Musculoskeletal conditions are a leading cause of disability, yet functioning is not routinely documented in primary care. A setting-specific International Classification of Functioning, Disability and Health (ICF) Core Set may facilitate biopsychosocial assessment by identifying domains relevant to patients and physical therapists.

**Objective:**

To empirically evaluate category retention and perceived relevance within a specific ICF Core Set for musculoskeletal conditions in primary care physical therapy and examine correspondence and differences between patient-reported and physical therapist-assessed perspectives.

**Methods:**

A multicenter, cross-sectional study included 387 patient-physical therapist dyads from the public regional healthcare service of Castilla y León (SACYL), Spain. Both perspectives rated 34 ICF categories using a 100 mm VAS. ICF categories with ≥5% prevalence were retained. Sensitivity analyses examined retention across WHODAS 2.0 and Self-administered Comorbidity Questionnaire (SCQ) levels and their continuous associations with ICF summary ratings. Paired perspectives were compared using Wilcoxon signed-rank tests with Holm-adjusted *p*-values, effect sizes, ICC(2,1), and individual absolute differences exceeding an interpretive 15 mm benchmark. The study was reported according to STROBE.

**Results:**

All 34 ICF categories exceeded the retention threshold (23.3–94.3%) and remained retained for all disability and comorbidity subgroups. WHODAS 2.0 showed moderate positive correlations with Body Functions and Activities and Participation ratings (Spearman's *ρ* = 0.329–0.357), whereas SCQ correlations were weaker (*ρ* = 0.178–0.202). Absolute paired-score correspondence ranged from moderate to excellent (ICC = 0.697–0.958). Twenty-seven comparisons were significant before adjustment and 20 after Holm adjustment. Differences were generally small (|*r*| = 0.012–0.356); the largest mean difference was 7.83 mm for “b710 Mobility of joint functions”. Across categories, 5.7%–29.3% of dyads exceeded 15 mm.

**Discussion:**

The findings provide empirical support for retaining the 34 categories and indicate generally substantial group-level correspondence alongside potentially informative individual differences. Further research should evaluate measurement properties, feasibility, responsiveness, and implementation in routine primary care.

## 1 Introduction

Disability has become a key global health issue due to a significant epidemiological shift towards non-communicable diseases, which now account for nearly two-thirds of worldwide years lived with disability (YLDs) (1). Musculoskeletal conditions are particularly relevant, affecting over 1.7 billion people, constituting the leading cause of disability and representing approximately 17% of all global years lived with disability (2). In this context, traditional health indicators are insufficient to capture the patient's lived experience of the disease (3), highlighting the growing importance of assessing functioning, and making its systematic measurement essential within rehabilitation settings (4).

Musculoskeletal conditions are characterized by pain and functional limitations. They have also been associated with psychological conditions and chronic pain, which impair quality of life and contribute to systemic multimorbidities (5). These factors exert a profound negative impact on the patient's personal, occupational, and social spheres (6). Consequently, timely rehabilitation is crucial, as delayed management has been shown to be detrimental to long-term patient outcomes (7). Community-based rehabilitation (CBR) services can provide strategies to prevent disability and enhance functioning in patients with musculoskeletal disorders (8). Patient-centered physical therapy interventions, such as education and therapeutic exercise, have been shown to be both effective and cost-effective (9–11).

However, measuring functioning remains a significant challenge across health disciplines and healthcare systems, complicating the implementation of the biopsychosocial framework in routine clinical practice (12). In response, the World Health Organization (WHO) proposed the International Classification of Functioning, Disability and Health (ICF), which provides a standardized method for recording functioning data. The ICF provides a neutral language and a conceptual framework that facilitate communication across health disciplines and throughout the continuum of care. According to the ICF, functioning is conceptualized as the dynamic result of the interaction between health conditions and contextual factors. The ICF operationalizes the biopsychosocial model by linking these domains through a comprehensive system of categories and qualifiers (13).

Incorporating the ICF framework into CBR services is crucial, yet its successful adoption in clinical practice depends heavily on the availability of facilitating tools (14). To address this operational need, ICF Core Sets have been developed to improve the clinical applicability of the classification (15). An ICF Core Set is a tailored selection of categories designed to describe patient functioning within specific health conditions or clinical settings. Two ICF Core Sets exist for musculoskeletal conditions, covering the acute and post-acute phases (16, 17). The Comprehensive ICF Core Set for post-acute musculoskeletal conditions includes 70 categories: 23 Body Functions, 7 Body Structures, 22 Activities and Participation categories, and 18 Environmental Factors. Its evaluation in Spanish primary care physical therapy services supported the relevance of the original categories but also identified limitations regarding its length, clinical feasibility, and coverage of aspects of functioning particularly relevant to physical therapy practice (18–21).

Based on these findings, a shorter, setting-specific ICF Core Set comprising 42 categories was proposed for primary care physical therapy (22). The present study focused on the 34 categories belonging to Body Functions (b), Activities and Participation (d) categories, and Environmental Factors (e), comprising 17, 10 and 7 categories, respectively. Of these 34 categories, 28 were retained from the comprehensive ICF Core Set for post-acute musculoskeletal conditions, whereas six additional categories were incorporated. The added Body Functions (b) categories expanded the assessment of sensory and sensorimotor functioning (“b235 Vestibular functions”, “b240 Sensations associated with hearing and vestibular function”, and “b265 Touch function”) and strengthened the description of movement-related functioning through “b720 Mobility of bone functions” and “b750 Motor reflex functions”. These domains are particularly relevant to physical therapy because the assessment and management of the movement system require consideration of sensory integration, postural control, joint and muscle function, and motor control (23). Finally, “d999 Community, social and civic life, unspecified” was included to document relevant activity limitations or participation restrictions not adequately represented by the other selected categories. The categories not retained mainly addressed broader physiological, self-care, attitudinal, and environmental aspects that were considered less directly aligned with the scope of a generic assessment of musculoskeletal functioning in primary care physical therapy (22).

Taken together, these modifications resulted in a specific ICF Core Set designed to reflect the scope and practical requirements of primary care physical therapy services while remaining applicable across the range of musculoskeletal conditions managed in this setting. However, the empirical retention and perceived relevance of the included ICF categories have not yet been formally evaluated, and the correspondence and differences between patient-reported and physical therapist-assessed perspectives have not yet been examined. These issues are important for documenting functioning within the ICF framework (24), and for developing specific ICF-based tools for this purpose, such as Clinical Assessment Schedules (CLAS) (25).

This study was framed around two complementary aims. First, we aimed to empirically evaluate the prevalence and perceived relevance of the categories included in the specific ICF Core Set for the management of musculoskeletal conditions in primary care physical therapy services in Spain. Second, we aimed to examine the correspondence and differences between patient-reported and physical therapist-assessed ratings across the selected ICF categories.

## 2 Methods

### 2.1 Study design

To empirically evaluate the prevalence and perceived relevance of the categories included in the specific ICF Core Set for the management of musculoskeletal conditions, a multicenter, cross-sectional study was conducted within the primary care physical therapy services of the Public Healthcare Service of Castilla y León (SACYL), Spain. This study was conducted and reported in accordance with the STrengthening the Reporting of OBservational studies in Epidemiology (STROBE) statement for cross-sectional studies (26). STROBE was used as a reporting guideline for the observational design, not as a framework for psychometric content validity assessment.

### 2.2 Setting

SACYL is a publicly funded regional healthcare system operating under a direct public management model that provides comprehensive healthcare services to a population of approximately 2.4 million inhabitants. CBR is delivered within this system through a coordinated network of primary care physical therapy units that provide direct support to primary care teams. Community-based physical therapy services are linked to the territory through a decentralized distribution across specific local healthcare districts. These services are composed of physical therapists who are primarily responsible for the management and rehabilitation of musculoskeletal conditions within the general population.

To establish the network of evaluators, a system-wide open call was issued across all health areas to recruit physical therapists as voluntary research collaborators. Of those invited, a total of 71 physical therapists agreed to participate. Prior to data collection, all collaborating therapists underwent standardized training regarding the ICF framework, as well as the specific design and objectives of the study. Following this process, a total of 47 primary care physical therapy units successfully contributed data to the study. The reasons why some initially interested therapists did not ultimately contribute data were not systematically recorded; this mainly reflects the pragmatic and voluntary nature of the multicenter recruitment process.

The study protocol was reviewed and granted ethical approval by the Clinical Research Ethics Committee of the Burgos and Soria health areas on February 27, 2024 (under reference code CEIm 3096). Additionally, the study protocol was prospectively registered in ClinicalTrials.gov under the identifier NCT06326229 (available at: https://clinicaltrials.gov/study/NCT06326229). Registration was performed for transparency and traceability and does not imply that the study had an interventional trial design.

Patient recruitment commenced on June 17, 2024, and was completed on May 22, 2025. To ensure participant privacy and data protection, all clinical data were pseudonymized by the collaborating physical therapists at the primary care units before transmission. Coded datasets were then forwarded to the principal investigator for analysis; consequently, the principal investigator remained completely blinded to the participants' identities throughout the entire study.

### 2.3 Participants

Study participants were selected from patients with musculoskeletal conditions who were referred for physical therapy by primary care physicians.

The inclusion criteria were as follows: (a) patients over 18 years of age; (b) a formal diagnosis of a musculoskeletal disorder by a primary care physician; and (c) sufficient proficiency in the Spanish language to complete the evaluations.

Conversely, patients were excluded: (a) if they were referred for a non-musculoskeletal condition (e.g., neurological disorders); or (b) if their physical therapy referral originated from hospital-based specialized services rather than primary care. Eligibility was therefore determined by the origin of the referral pathway, in order to preserve the primary care context of the study.

All eligible patients were thoroughly informed about the study's purpose, design, and procedures, and they were invited to participate voluntarily. Potential participants were given the opportunity to ask questions, and those who agreed to participate provided written informed consent prior to any data collection. This entire process and the study protocol were conducted in accordance with the Declaration of Helsinki (October 2024).

### 2.4 Variables

The primary variable of interest for this study was the empirical prevalence and perceived relevance of the categories included in the specific ICF Core Set for the management of musculoskeletal conditions in primary care physical therapy services (22), when applied in the assessment of patients suffering from musculoskeletal conditions.

As a secondary variable, the degree of correspondence between patients and physical therapists regarding the relevance of these ICF categories was examined to assess convergence and divergence between the patient's self-report and the clinician's professional evaluation.

Finally, sociodemographic and clinical variables were collected to characterize the sample, including age, sex, educational level, occupation, musculoskeletal diagnosis, disability status, presence of comorbidities, and health-related quality of life.

### 2.5 Outcome measures

#### 2.5.1 Tailored ICF-based data collection form

The specific ICF Core Set for the management of musculoskeletal conditions in primary care physical therapy services was empirically evaluated using an ICF-based standardized form developed from the ICF-based Documentation Tool (available at: https://www.icf-core-sets.org; accessed on June 12, 2024).

This purpose-designed form presented the categories included in the specific ICF Core Set along with their definitions. This ICF Core Set is composed of a total of 42 ICF categories (8 from the “body structures” component, 17 from “body functions”, 10 from “activities and participation”, and 7 from “environmental factors”) and 14 personal factors (22). The ICF categories corresponding to the “body structures” component were not included in the standardized form, as the inclusion criteria did not restrict participants by specific anatomical lesion sites or specific pathological locations.

A 100 mm visual analog scale (VAS) was integrated into the form to independently rate the degree to which each ICF category was perceived by patients or assessed by physical therapists as relevant to functioning. The VAS scores ranged from 0 (“no problem”) to 100 mm (“total problem”). Visual analog scales are widely used to quantify subjective experiences that are not directly observable, such as pain or perceived intensity (27). In the present study, the VAS was used as a category-specific rating procedure to quantify the degree to which each ICF category was perceived by patients or assessed by physical therapists as a functioning-related problem. Therefore, VAS scores were used to estimate prevalence and rating magnitude, rather than to infer formal ICF severity qualifiers, establish a new ICF qualifier conversion system, or demonstrate the psychometric validity of the Core Set.

#### 2.5.2 World health organization disability assessment schedule 2.0 (WHODAS 2.0)

Patient disability status and functional limitations were evaluated using the Spanish version of the World Health Organization Disability Assessment Schedule 2.0 (WHODAS 2.0) (28). WHODAS 2.0 is conceptually aligned with the International Classification of Functioning, Disability and Health (ICF) and evaluates functioning across major life domains. Its development involved cross-cultural field studies supporting its applicability, reliability, validity, and usefulness in health services research (29). This instrument provides a standardized measure of functioning and disability across six distinct life domains: cognition (understanding and communicating), mobility (moving and getting around), self-care (hygiene, dressing, and eating), getting along (interacting with other people), life activities (domestic responsibilities, leisure, and work), and participation (joining in community activities). All items are scored on a 5-point Likert scale. Scores were computed using the simple scoring method, which involves direct summation of item responses based on classical test theory, followed by linear transformation to a 0–100 scale. Previous evidence supports its cross-cultural applicability, reliability, validity, and usefulness in health services research, with the original development study reporting high internal consistency (Cronbach's alpha = 0.86) and excellent test-retest reliability (intraclass correlation coefficient = 0.98) (30). More recent systematic evidence also supports the reliability of WHODAS 2.0, reporting excellent internal consistency for the 36-item version and good reliability for the 12-item version (31).

#### 2.5.3 World health organization quality of life brief version (WHOQOL-BREF)

Health-related quality of life was assessed using the Spanish version of the WHOQOL-BREF questionnaire (32). This validated, 26-item self-report instrument evaluates quality of life across four primary domains: physical health (7 items), psychological health (6 items), social relationships (3 items), and environmental health (8 items), along with two standalone items measuring overall quality of life and general health. All items are rated on a 5-point Likert scale. The Spanish WHOQOL-BREF has shown satisfactory psychometric properties, including acceptability, internal consistency, and evidence of convergent and discriminant validity. In a Spanish sample of older adults, the WHOQOL-BREF showed high internal consistency, with Cronbach's alpha = 0.90 for the overall scale (33). Raw domain scores were calculated and linearly transformed into a normalized scale ranging from 0 to 100, where higher scores reflect a better perceived quality of life.

#### 2.5.4 Self-administered comorbidities questionnaire (SCQ)

Comorbidities were evaluated using the Spanish version of the Self-Administered Comorbidities Questionnaire (SCQ) (34). This self-report instrument assesses the presence, treatment, and functional impact of 12 predefined chronic medical conditions. For each condition, a maximum of 3 points can be assigned: 1 point for the presence of the problem, 1 point if the patient receives medical treatment for it, and 1 point if the condition limits their daily activities. The total score is calculated by summing the points across all conditions, yielding a scoring range from 0 to 36 for the closed-ended items, where higher scores represent a greater cumulative burden of chronic disease on functional status. The SCQ was developed as an efficient method for assessing comorbidity in clinical and health services research, particularly when medical record abstraction is not feasible. In the original validation study, the SCQ showed modest correlation with the Charlson Index (Spearman *r* = 0.32), which increased when comparable items were considered (Spearman *r* = 0.55), and showed associations with subsequent health status and health care utilization (35).

#### 2.5.5 Sociodemographic data form

A purpose-designed sociodemographic and clinical questionnaire was used to characterize the study sample. This form was completed at baseline and recorded the following participant variables: (a) age (measured in years), (b) sex (documented for compliance with sex and gender equity guidelines), (c) educational level (categorized by highest degree attained), (d) current occupation or employment status, and (e) the primary musculoskeletal diagnosis that motivated the referral to physical therapy service.

### 2.6 Procedures

The method for collecting data was based on semi-structured interviews conducted by the collaborating physical therapists as part of the routine assessment of patients referred for musculoskeletal conditions.

In the first part of the interview, sociodemographic and clinical data were collected by the physical therapists using the standardized form described previously to characterize the sample and to confirm that all eligibility criteria were met.

Subsequently, the tailored ICF-Based Data Collection Form was used to collect information on functioning-related problems. The physical therapists presented the list of ICF categories to the patients and asked them to assess their relevance using the VAS. The role of the physical therapists was to ensure that patients thoroughly understood the concepts involved in each ICF category. In this way, the form was completed by the patient under the supervision of the physical therapist, who was available to clarify any questions. Detailed instructions on how to conduct this process correctly had been provided to physical therapists during the prior standardized training session.

Thereafter, the physical therapists performed their routine clinical assessment of the patient and independently completed the identical ICF-based data form from their own professional perspective. Finally, following the clinical assessment, patients were provided with the self-administered questionnaires described in the previous section (SCQ, WHODAS 2.0, and WHOQOL-BREF). Patients were given one week to complete these questionnaires at home and return them to their responsible physical therapist.

### 2.7 Potential sources of bias

To ensure the internal validity of the study, several strategies were implemented to address potential sources of bias.

First, to minimize selection bias and enhance sample representativeness, a multicenter approach was adopted by recruiting participants from multiple independent primary care physical therapy units across different healthcare districts within the SACYL.

Second, to control for information and measurement bias, the collaborating physical therapists underwent standardized training prior to the study to ensure uniform criteria during the data collection process. Physical therapists were not fully blinded to the patient interview process because they supervised patients to clarify the conceptual definitions of the ICF categories. However, the professional evaluation was recorded separately and was based on the therapist's routine clinical assessment. This procedure was intended to minimize the influence of patient self-reported scores on the therapist's professional judgment.

Third, to mitigate detection and analyzer bias, all datasets were completely pseudonymized at the clinical source; the principal investigator received exclusively coded data and remained fully blinded to the identities of both patients and therapists throughout the entire data processing and statistical analysis stages.

Finally, sensitivity analyses were conducted to examine whether category retention remained stable across broad levels of disability (assessed via WHODAS 2.0) and comorbidity burden (assessed via SCQ).

### 2.8 Study size and sampling method

The sample size was set at 385 patients. This was calculated considering a finite target population of 2,040,153 inhabitants aged 18 years or older in the autonomous community of Castilla y León, Spain, according to 2022 data from the Spanish National Statistics Institute (*Instituto Nacional de Estadística, INE*). The calculation was performed assuming a 95% confidence level, a maximum variability or expected proportion of 50% (*p* = 0.5), and a margin of error of ±5%.

A non-probability consecutive sampling method was utilized to recruit participants. All patients with musculoskeletal conditions who met the eligibility criteria and attended the primary care physical therapy services during the study period were sequentially invited to participate. The sample size calculation was used to define a target sample adequate for estimating the prevalence of ICF categories with acceptable precision. However, because recruitment was based on consecutive non-probability sampling rather than random population sampling, this calculation should not be interpreted as establishing population representativeness.

### 2.9 Operationalization of quantitative variables

The empirical evaluation of the ICF Core Set was conducted through a prevalence analysis of functioning-related problems, established strictly based on the limitations experienced and reported by the patients. This prevalence-based approach was used as an empirical category-retention strategy within the methodological tradition of ICF Core Set studies (36). It should be distinguished from content validity as a psychometric measurement property, which additionally requires explicit assessment of relevance, comprehensiveness, and comprehensibility (37).

The ICF-based standardized form collected quantitative data via a VAS to capture the degree ofperceived or professionally assessed relevance attributed to each specific ICF category. Raw quantitative scores were subsequently operationalized for prevalence analysis according to predefined thresholds. Qualifier 0 was defined as “no problem” (VAS scores less than 5 mm), whereas qualifiers 1–4 were defined as “presence of a problem” (VAS scores from 5 to 100 mm).

Qualifiers 8 (not specified) and 9 (not applicable) were conceptually considered distinct from qualifier 0 (“no problem”). Therefore, they were not interpreted as equivalent to absence of a problem. However, for the purpose of the prevalence analysis, categories coded as 8 or 9 were not counted as confirmed presence of a functioning-related problem. This analytical decision was made to avoid overestimating category prevalence when the category could not be sufficiently specified or was not applicable to the patient's situation, and it was considered when interpreting the findings.

Consequently, individual category ratings were aggregated to estimate the prevalence of functioning-related problems for each ICF category. The original continuous VAS ratings were used to examine correspondence and differences between patient-reported and physical therapist-assessed perspectives.

### 2.10 Statistical methods

The statistical methods employed for the analysis of the study variables are presented in detail in the following subsections.

#### 2.10.1 Descriptive and sociodemographic analysis

Descriptive statistics were used to characterize the sociodemographic and clinical profile of the study sample. Quantitative continuous variables (including age, WHODAS 2.0, WHOQOL-BREF, and SCQ) were expressed as means and standard deviations (SD) or medians and interquartile ranges (IQR), depending on data normality. Data normality was formally assessed using the Kolmogorov–Smirnov test. Categorical variables, such as sex, educational level, occupation, and primary musculoskeletal referral diagnosis, were expressed as absolute frequencies and percentages.

#### 2.10.2 Empirical evaluation of the ICF core set categories

Descriptive statistics were used to analyze the primary variable of interest. For the empirical evaluation of the ICF Core Set categories, the prevalence of functioning-related problems was calculated for each individual ICF category by determining the relative frequency (percentage) of patients whose ratings indicated the presence of a problem. For Body Functions (b) and Activities and Participation (d), VAS scores below 5 mm were categorized as “no problem”, whereas scores from 5 to 100 mm were categorized as “presence of a problem” for prevalence estimation.

Environmental Factors (e) were interpreted separately from Body Functions and Activities and Participation. In accordance with the ICF framework, Environmental Factors (e) may act either as barriers or as facilitators to functioning, depending on the person's context. Therefore, in the present study, Environmental Factors (e) were not interpreted as “problems” in the same sense as body impairments or activity limitations. Rather, they were considered contextual factors perceived by patients or assessed by physical therapists as relevant to functioning. The prevalence estimates for this component consequently represent the frequency with which each Environmental Factor (e) was identified as relevant, rather than the prevalence of environmental problems, barriers, or facilitators. The study did not distinguish whether each Environmental Factor acted as a barrier or as a facilitator, and this distinction was considered when interpreting the findings.

An individual ICF category was considered retained within the Core Set if it was identified as a problem, or as a relevant contextual factor in the case of Environmental Factors, by at least 5% of the study sample. The 5% value was used as a predefined empirical category-retention criterion and was not interpreted as a complete assessment of content validity as a psychometric measurement property.

#### 2.10.3 Sensitivity analyses according to disability and comorbidity burden

Sensitivity analyses were conducted to examine whether the retention of the ICF categories remained stable across different levels of disability and comorbidity burden. First, the study sample was stratified using the observed medians of the WHODAS 2.0 and SCQ total scores. For each subgroup, the prevalence or frequency of relevance of every ICF category was recalculated, and the predefined 5% retention criterion was reapplied. Because dichotomization of continuous variables may result in loss of information, the median-based subgroup analyses were interpreted only as pragmatic sensitivity analyses of category-retention stability. They were not intended to estimate graded, explanatory, or causal associations between disability, comorbidity burden, and ICF category ratings.

To preserve the continuous nature of WHODAS 2.0 and SCQ scores, complementary Spearman rank correlation analyses were performed. Patient-reported VAS scores were summarized by calculating mean ratings for Body Functions, Activities and Participation, the combined functioning components of Body Functions and Activities and Participation, and Environmental Factors. A component summary score was calculated when at least 80% of its constituent category ratings were available; otherwise, the summary score was treated as missing.

WHODAS 2.0 and SCQ total scores were correlated separately with each of the four ICF summary ratings. Environmental Factors were analyzed and interpreted separately because their ratings represented contextual relevance and did not distinguish whether the factors acted as barriers or facilitators. Spearman correlation coefficients were reported with percentile bootstrap 95% confidence intervals based on 5,000 participant-level resamples. To address the potential inflation of Type I error, *p*-values were adjusted using the Holm procedure within each family of four correlations. Statistical tests were two-tailed, with the significance level set at *α* = 0.05.

#### 2.10.4 Correspondence between patient-reported and physical therapist-assessed perspectives

To analyze the secondary variable of interest, the degree of correspondence and potential discrepancies between the continuous VAS scores (0–100 mm) assigned within each patient-physical therapist dyad were evaluated for each ICF category. Patient and physical therapist ratings were compared separately across the 34 ICF categories using the Wilcoxon signed-rank test for paired observations. Differences were calculated as the patient rating minus the physical therapist rating; therefore, positive values indicated higher patient ratings, whereas negative values indicated higher physical therapist ratings. For each category, only dyads with valid ratings from both the patient and the physical therapist were included, and the number of valid pairs was reported.

To address the potential inflation of Type I error arising from multiple comparisons, unadjusted *p*-values were supplemented with *p*-values adjusted across the 34 comparisons using the Holm procedure. All tests were two-tailed, with the statistical significance level set at *α* = 0.05.

Patient-reported and physical therapist-assessed VAS ratings were summarized separately using means and standard deviations and medians and interquartile ranges. The magnitude and direction of patient-physical therapist differences were described using mean and median paired differences. Mean paired differences were reported with their percentile bootstrap 95% confidence intervals, estimated using 10,000 participant-level resamples. Effect sizes for the Wilcoxon signed-rank test were calculated as *r* = |*Z*|/√*N*, where |*Z*| was the absolute value of the standardized test statistic and *N* was the number of valid paired observations for each category. The direction of the differences was determined from the paired difference, calculated as patient rating minus physical therapist rating, rather than from the sign of the Wilcoxon Z statistic. Effect sizes were interpreted descriptively, with absolute values of approximately 0.10, 0.30, and 0.50 indicating small, moderate, and large effects, respectively.

Absolute correspondence between paired patient and physical therapist ratings was additionally summarized using a two-way random-effects, absolute-agreement, single-measure intraclass correlation coefficient (ICC[2,1]). Percentile bootstrap 95% confidence intervals for the ICC estimates were calculated using 10,000 dyad-level resamples. ICC values were interpreted as poor (<0.50), moderate (0.50–0.75), good (0.75–0.90), or excellent (>0.90) based on established guidelines (38). Because patients and physical therapists represent complementary perspectives rather than interchangeable raters, ICC values were interpreted cautiously as indicators of absolute correspondence between paired scores rather than as evidence of inter-rater reliability in the strict sense.

To complement group-level comparisons, individual-level discrepancies were examined by calculating the absolute difference between patient and physical therapist ratings within each dyad. The number and proportion of dyads with absolute differences greater than 15 mm were calculated for each category. The predefined 15-mm value was used only as a conservative interpretive benchmark for potentially meaningful between-perspective differences (39) and was not considered a minimal clinically important difference or a formally validated cutoff for patient-professional disagreement.

#### 2.10.5 Missing data

Missing data were handled using an available-case approach, without imputation. Participants with missing values for a specific variable were excluded only from the analysis involving that variable. Prevalence estimates were calculated using the number of valid observations available for each ICF category as the denominator. For paired patient-physical therapist comparisons, only dyads with valid ratings from both participants were included.

#### 2.10.6 Software and data management

All statistical computations were executed using SPSS 29.0 for Windows (IBM, Armonk, NY, USA), and Python, version 3.12.9 (Python Software Foundation, Wilmington, DE, USA). SPSS was used for the initial descriptive and inferential analyses, whereas Python was used for complementary analyses, including multiple-comparison adjustments, effect-size calculations, percentile bootstrap confidence intervals, individual-level absolute-difference analyses, and continuous-variable sensitivity analyses. The datasets generated and analyzed during the current study are stored by the principal investigator and are available from the corresponding author upon reasonable request.

## 3 Results

A total of 387 patients with musculoskeletal conditions successfully participated in the study, with valid case distribution per variable detailed in Table 1. The mean age of the participants was 57.8 ± 13.6 years (range: 20–90 years), with a sex distribution showing a clear predominance of women (67.8%). The most frequent primary reasons for clinical referral from primary care physicians were low back pain (24.3%) and upper limb tendinopathies (16.5%), followed by neck pain and lower limb osteoarthritis (both at 12.4%).

**Table 1:** Baseline sociodemographic, clinical, and health-related characteristics of the sample.

Variable	Full sample (*N* = 387)	Missing data
Sociodemographic characteristics		
Age (years), *mean* (*SD*)	57.8 (13.6)	
Sex, *n* (%)		2
Female	261 (67.8)	
Male	124 (32.2)	
Educational Level, *n* (%)		4
No formal education	11 (2.9)	
Primary or secondary education	142 (37.1)	
Vocational training	94 (24.5)	
Upper secondary education	63 (16.4)	
Higher education	69 (18.0)	
Other	4 (1.0)	
Employment Status, *n* (%)		4
Student	3 (0.8)	
Unemployed	29 (7.6)	
Active worker	196 (51.2)	
Retired	123 (32.1)	
Disabled for work	7 (1.8)	
Other	25 (6.5)	
Urban-rural status		
Urban	228 (58.9)	
Rural	159 (41.1)	
Primary clinical referral diagnosis, *n* (%)		2
Neck pain	48 (12.5)	
Low back pain	94 (24.4)	
Ankle sprain	6 (1.6)	
Shoulder pain	35 (9.1)	
Upper limb tendinopathies	64 (16.6)	
Lower limb tendinopathies	39 (10.1)	
Upper limb osteoarthritis	21 (5.5)	
Lower limb osteoarthritis	48 (12.5)	
Other	30 (7.8)	
Clinical and functional status		
Presence of comorbidities (SCQ score, 0–36)		4
Mean (SD)	5.1 (4.1)	
Median [IQR]	4.0 [5.0]	
Functional Disability Status (WHODAS 2.0 global score, 0–100)		23
Mean (SD)	21.0 (16.8)	
Median [IQR]	16.7 [20.8]	
Health-related quality of life (WHOQOL-BREF)		
Standalone Global Items (0–100 scale)		
Overall quality of life, *median [IQR]*; *mean (SD)*	50.0 [50.0]; 52.5 (23.5)	5
Satisfaction with own health, *median [IQR]; mean (SD)*	50.0 [25.0]; 43.8 (23.5)	5
Specific Domains (0–100 scale)		
Physical health, *mean (SD)*	53.5 (18.3)	14
Psychological health, *median [IQR]; mean (SD)*	62.5 [20.8]; 60.3 (17.0)	13
Social relationships, *median [IQR]*; *mean (SD)*	46.7 [20.0]; 48.2 (15.1)	21
Environmental health, *mean (SD)*	47.9 (11.2)	18

IQR, interquartile range; n, number of patients; SD, standard deviation; SCQ, self-administered comorbidities questionnaire; WHODAS 2.0, World Health Organization disability assessment schedule 2.0; WHOQOL-BREF, World Health Organization quality of life brief version.

Variable display formatting [Mean (SD) vs. Median [IQR]] was selected based on normal and non-normal distribution data confirmed by the Kolmogorov–Smirnov test.

Regarding functional status and potential clinical confounders, the sample exhibited a median baseline disability score of 16.7 [IQR: 20.8] on the WHODAS 2.0 (mean: 21.0 ± 16.8 points out of 100). Concerning cumulative comorbidity burden via the SCQ, patients displayed a median score of 4.0 [IQR: 5] points (mean: 5.1 ± 4.1; range: 0–30), reflecting high clinical heterogeneity.

Concerning health-related quality of life evaluated through standalone global items of the WHOQOL-BREF, the sample reported a median score of 50.0 [IQR: 50.0] on a 0–100 scale for overall perception of quality of life (mean ± SD: 52.5 ± 23.5) and a median score of 50.0 [IQR: 25.0] on a 0–100 scale for satisfaction with one's own health (mean ± SD: 43.8 ± 23.5). Furthermore, a detailed analysis of the four domains revealed that psychological health achieved the highest score [median: 62.5 (IQR: 20.8)], followed by physical health (mean ± SD: 53.5 ± 18.3). Conversely, the lowest scores were observed for social relationships [median: 46.7 (IQR: 20)] and environmental health (mean ± SD: 47.9 ± 11.2).

### 3.1 Empirical evaluation of the ICF core set categories

The prevalence of functioning-related problems, together with the mean scores and standard deviations for each evaluated ICF category, is presented in Table 2 and Figures 1–3. Notably, all 34 evaluated ICF categories exceeded the predefined 5% retention threshold, with prevalence rates ranging from 23.3% to 94.3%. No responses were coded as qualifier 8 (“not specified”) or qualifier 9 (“not applicable”); therefore, these qualifiers did not affect the prevalence estimates and no sensitivity analysis excluding or retaining these categories was required. Consequently, all evaluated categories were retained within the proposed specific ICF Core Set. These findings provide empirical support for the prevalence and perceived relevance of the included categories in this clinical context.

**Table 2:** Frequency distribution, relevance, and missing data of the specific ICF core set categories (*N* = 387).

ICF category	Description	Patient-reported relevance*		Missing data, *n*
		Problem (≥ 5 mm VAS) *n* (%)	No problem (< 5 mm VAS) *n* (%)	
b235	Vestibular functions	160 (41.3)	227 (58.7)	0
b240	Sensations associated with hearing and vestibular function	130 (33.6)	257 (66.4)	0
b260	Proprioceptive function	172 (44.6)	214 (55.4)	1
b265	Touch function	144 (37.2)	243 (62.8)	0
b270	Sensory functions related to temperature and other stimuli	118 (30.5)	269 (69.5)	0
**b280**	**Sensation of pain**	**365** **(94.3)**	**22** **(5.7)**	**0**
**b455**	**Exercise tolerance functions**	**286** **(73.9)**	**101** **(26.1)**	**0**
**b710**	**Mobility of joint functions**	**315** **(81.4)**	**72** **(18.6)**	**0**
b715	Stability of joint functions	188 (48.6)	199 (51.4)	0
**b720**	**Mobility of bone functions**	**223** **(57.6)**	**164** **(42.4)**	**0**
**b730**	**Muscle power functions**	**270** **(69.8)**	**116** **(30.2)**	**1**
**b735**	**Muscle tone functions**	**242** **(62.5)**	**145** **(37.5)**	**0**
**b740**	**Muscle endurance functions**	**268** **(69.3)**	**119** **(30.7)**	**0**
b750	Motor reflex functions	111 (28.7)	276 (71.3)	0
b760	Control of voluntary movement functions	127 (32.8)	259 (67.2)	1
**b770**	**Gait pattern functions**	**195** **(50.4)**	**191** **(49.6)**	**1**
**b780**	**Sensations related to muscles and movement functions**	**306** **(79.1)**	**81** **(20.9)**	**0**
**d410**	**Changing basic body position**	**239** **(61.8)**	**148** **(38.2)**	**0**
**d415**	**Maintaining a body position**	**234** **(60.5)**	**153** **(39.5)**	**0**
d420	Transferring oneself	172 (44.6)	214 (55.4)	1
**d430**	**Lifting and carrying objects**	**243** **(62.8)**	**144** **(37.2)**	**0**
d440	Fine hand use	110 (28.5)	276 (71.5)	1
d445	Hand and arm use	156 (40.4)	230 (59.6)	1
**d450**	**Walking**	**195** **(50.5)**	**191** **(49.5)**	**1**
d540	Dressing	163 (42.1)	224 (57.9)	0
d570	Looking after one's health	90 (23.3)	297 (76.7)	0
d999	Community, social and civic life, unspecified	148 (38.2)	239 (61.8)	0
**e110**	**Products or substances for personal consumption**	**238** **(61.7)**	**148** **(38.3)**	**1**
e120	Products and technology for personal indoor and outdoor mobility and transportation	165 (42.9)	220 (57.1)	2
**e310**	**Immediate family**	**260** **(67.5)**	**125** **(32.5)**	**2**
**e320**	**Friends**	**234** **(60.8)**	**151** **(39.2)**	**2**
e340	Personal care providers and personal assistants	117 (31.7)	252 (68.3)	18
**e355**	**Health professionals**	**309** **(79.8)**	**78** **(20.2)**	**0**
**e580**	**Health services, systems and policies**	**312** **(80.8)**	**74** **(19.2)**	**1**

ICF, Classification of Functioning, Disability and Health; VAS, Visual Analog Scale.

In bold: ICF categories that showed a prevalence over 50%.

* Percentages showed in this column represent the prevalence of the respective functioning domain. For body functions (b) and activities and participation (d), prevalence estimates indicate the presence of a functioning-related problem. For environmental factors (e), prevalence estimates indicate that the contextual factor was identified as relevant to functioning, without determining whether it acted as a barrier or facilitator.

**Figure 1:**
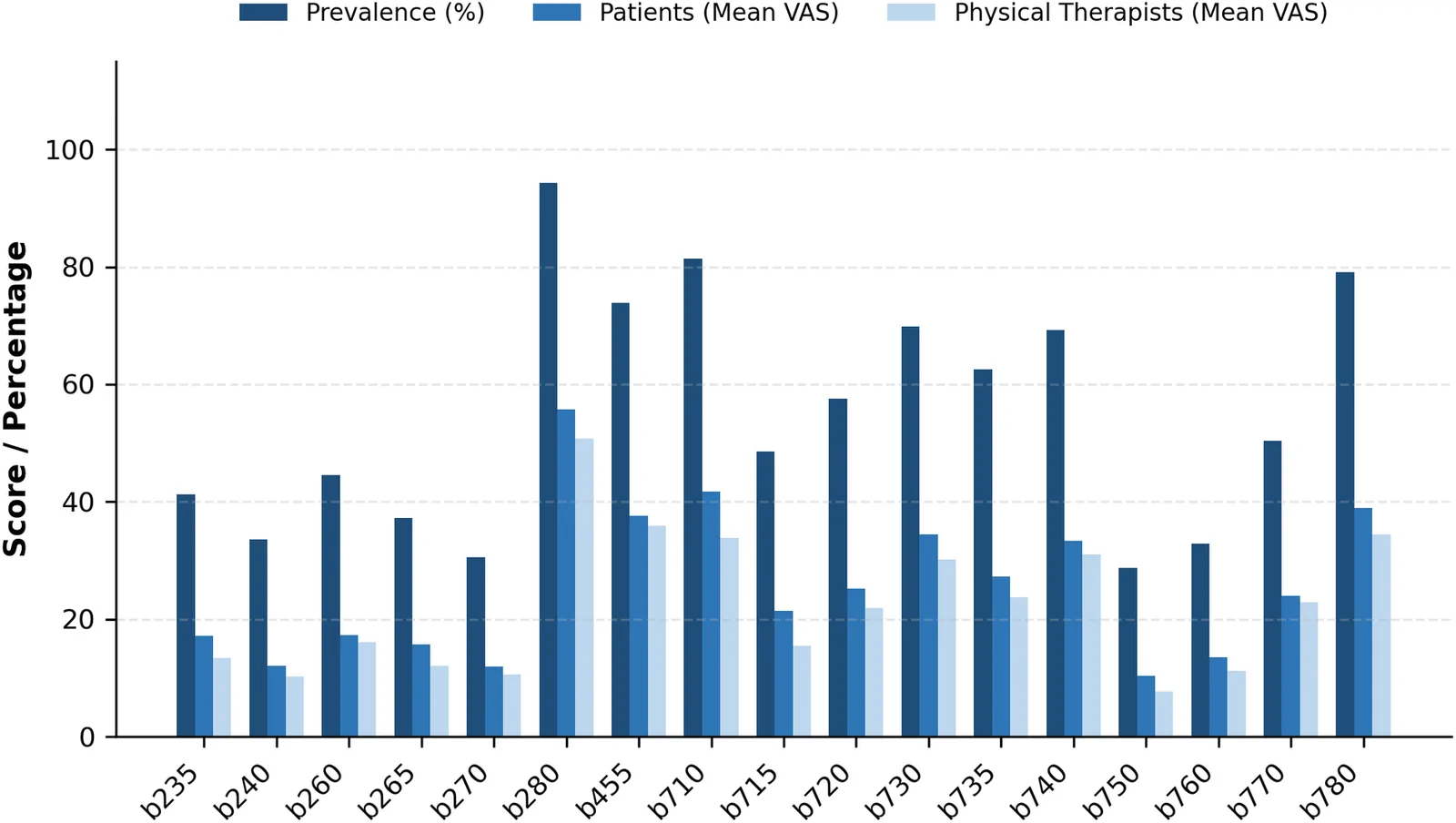
Prevalence and VAS ratings for Body Functions categories in the specific ICF Core Set from patient-reported and physical therapist-assessed perspectives.

**Figure 2:**
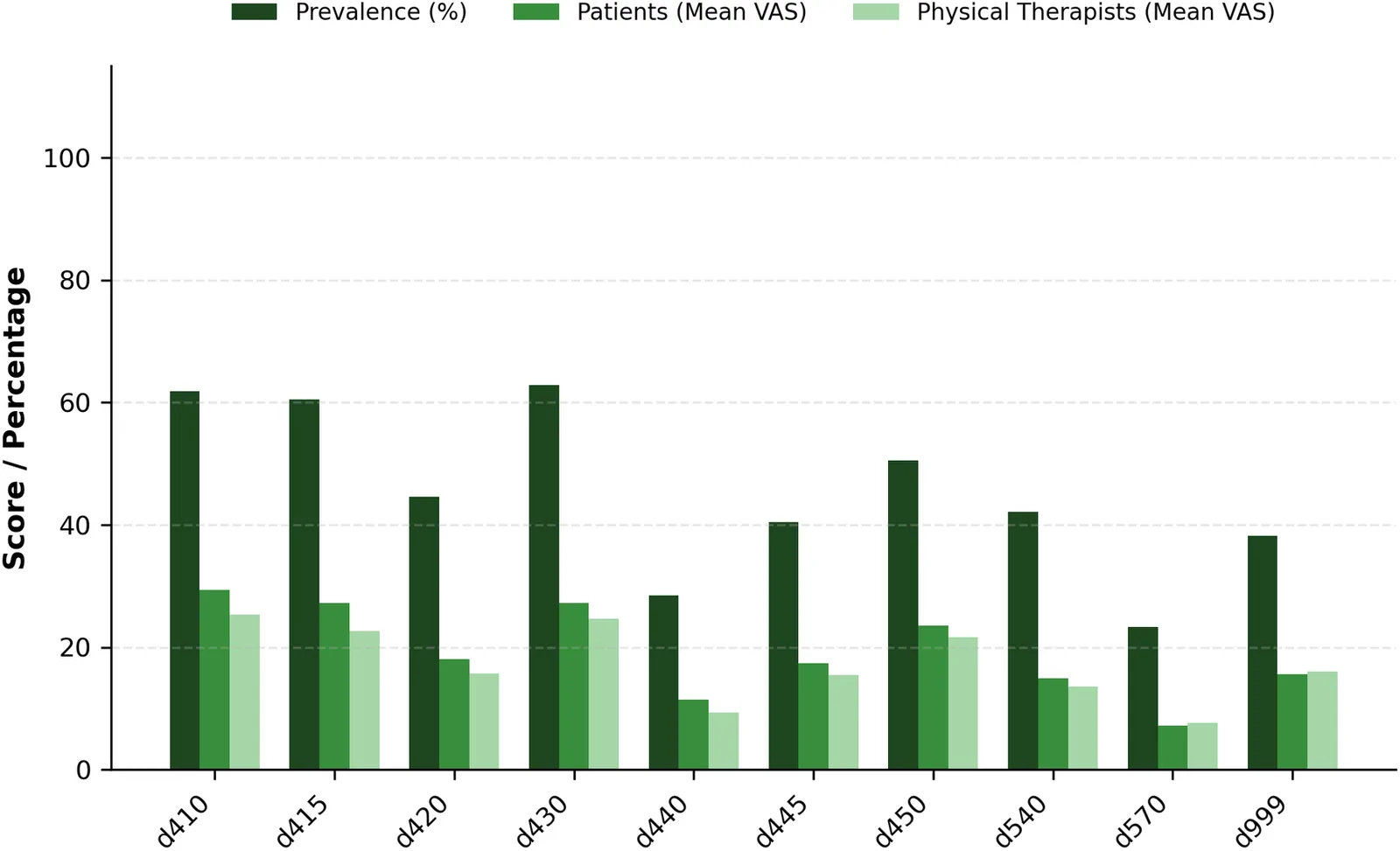
Prevalence and VAS ratings for Activities and Participation categories in the specific ICF Core Set from patient-reported and physical therapist-assessed perspectives.

**Figure 3:**
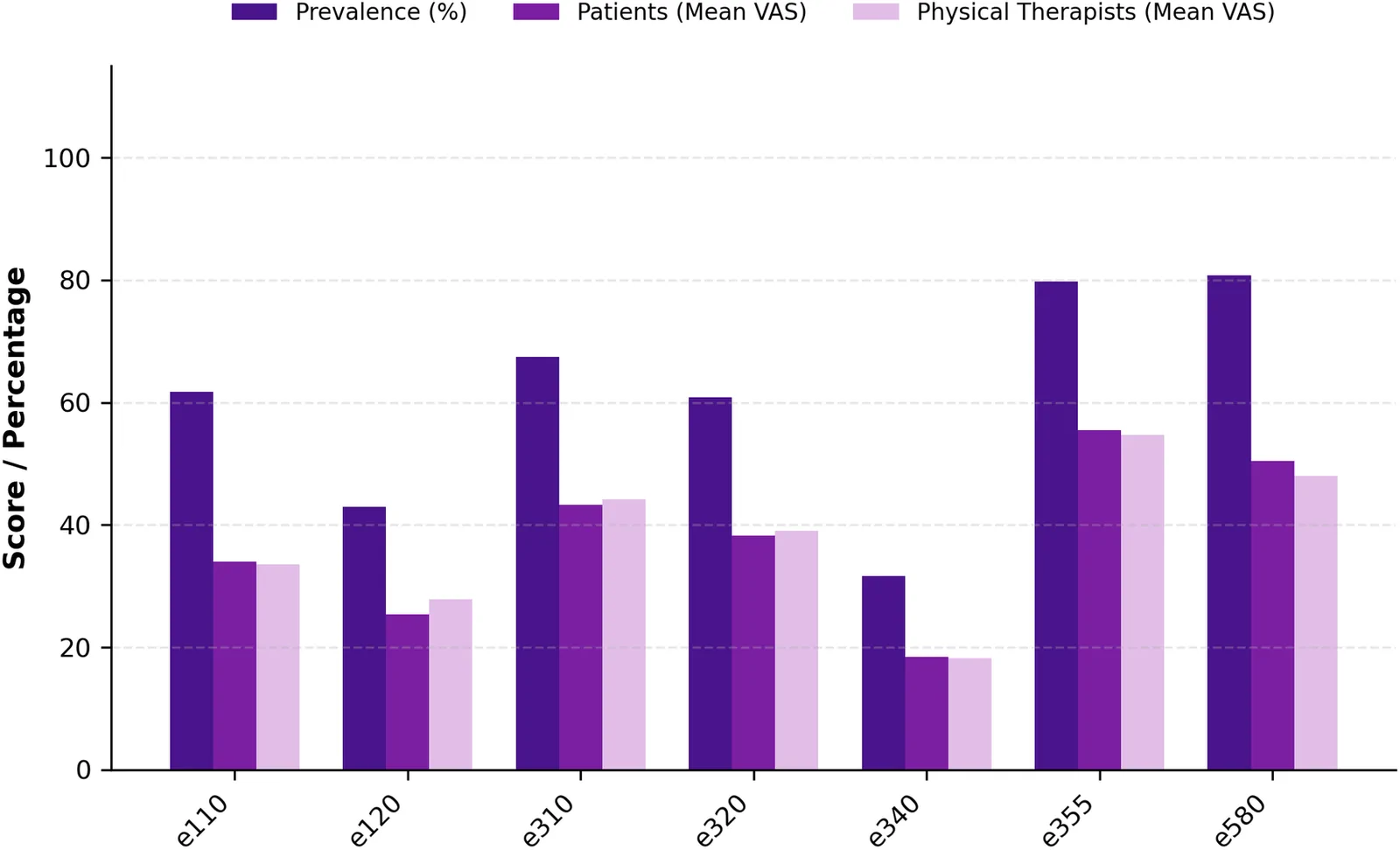
Prevalence and VAS ratings for Environmental Factors categories in the specific ICF Core Set from patient-reported and physical therapist-assessed perspectives.

Within the Body Functions (b) component, “b280 Sensation of pain” was identified as the most frequently reported functioning-related issue, exhibiting both the highest prevalence rate (94.3%) and the highest VAS score (55.7 ± 29.0 mm) across the entire sample. This was followed by a high frequency of impairments related to joint and muscle mechanics, specifically “b710 Mobility of joint functions” (81.4%, 41.7 ± 31.6 mm), “b780 Sensations related to muscles and movement functions” (79.1%, 39.0 ± 29.7 mm), and “b455 Exercise tolerance functions” (73.9%, 37.6 ± 31.8 mm). Conversely, “b750 Motor reflex functions” demonstrated the lowest prevalence within this component (28.7%, 10.3 ± 21.5 mm).

Regarding the Activities and Participation (d) component, the most prevalent functional limitations reported by patients were “d430 Lifting and carrying objects” (62.8%, 27.3 ± 30.2 mm) and “d410 Changing basic body position” (61.8%, 29.4 ± 30.4 mm). General mobility restrictions were further reflected by a 50.5% prevalence in “d450 Walking”. The least compromised activity domain was “d570 Looking after one”s health/parts of the body”, which nevertheless achieved a meaningful prevalence of 23.3% (7.2 ± 16.9 mm).

Finally, the Environmental Factors (e) component revealed a substantial perceived relevance of contextual elements, particularly within the healthcare environment. The highest prevalence rates were observed for “e580 Health services, systems, and policies” (80.8%, 50.5 ± 34.0 mm) and “e355 Health professionals” (79.8%, 55.5 ± 37.6 mm), followed by “e310 Immediate family” (67.5%, 43.3 ± 38.5 mm). In contrast, “e340 Personal care providers and personal assistants” presented the lowest relevance within this component (31.7%, 18.5 ± 32.5 mm). The prevalence estimates refer to the identification of contextual factors as relevant to functioning, rather than to the prevalence of problems, barriers, or facilitators. The study did not distinguish whether these factors had a facilitating or hindering effect.

### 3.2 Sensitivity analysis

The stability of the predefined 5% prevalence threshold for category retention was examined through sensitivity analyses. Stratification of the study sample based on the median split of the WHODAS 2.0 total score (median = 16.7) demonstrated that all 34 evaluated ICF categories consistently maintained prevalence rates well above the 5% cutoff in both the subgroup with scores at or below the median and the subgroup with scores above the median.

Similarly, when the sample was stratified by the median split of the cumulative comorbidity burden via the SCQ total score (median = 4.0), the retention status of the individual ICF categories remained unchanged across both subgroups. Even for category “d570 Looking after one”s health”, which exhibited the lowest baseline prevalence in the overall sample (23.3%), the subgroup prevalence estimates remained above the predefined threshold. These findings indicate that retention of the proposed specific ICF Core Set categories was stable across broad levels ofdisability and cumulative comorbidity burden (see Supplementary Table S1).

Complementary analyses preserving WHODAS 2.0 and SCQ as continuous variables showed positive associations with patient-reported ICF ratings. WHODAS 2.0 was moderately correlated with the mean VAS ratings for Body Functions (Spearman's *ρ* = 0.351; 95% CI: 0.253–0.447), Activities and Participation (*ρ* = 0.329; 95% CI: 0.226–0.430), and their combined summary score (*ρ* = 0.357; 95% CI: 0.257–0.452). SCQ showed weaker positive correlations with the corresponding ratings (*ρ* = 0.197, 0.178, and 0.202, respectively). All these associations remained statistically significant after Holm adjustment (see Supplementary Table S2).

Neither WHODAS 2.0 nor SCQ showed a statistically significant association with the Environmental Factors summary rating. These results were interpreted separately because Environmental Factor ratings reflected contextual relevance but did not indicate whether the factors acted as barriers or facilitators.

### 3.3 Correspondence between patient-reported and physical therapist-assessed perspectives

The analysis of paired patient-physical therapist perspectives across the 34 evaluated ICF categories is detailed in Tables 3, 4 (see also Figures 1–3). Absolute paired-score correspondence ranged from moderate to excellent across the evaluated ICF categories (ICC[2,1] range: 0.697–0.958]. These coefficients were interpreted descriptively because patients and physical therapists represent complementary perspectives rather than interchangeable raters. Using unadjusted *p*-values, statistically significant differences were identified in 27 of the 34 categories. After adjustment for multiple comparisons using the Holm procedure, 20 comparisons remained statistically significant. However, none of the group-level mean differences reached the predefined 15-mm interpretive benchmark. Absolute effect sizes ranged from 0.012 to 0.356, with a median of 0.180, indicating that the observed differences were generally small, although moderate effects were identified for a limited number of categories.

**Table 3:** Descriptive VAS ratings of functioning domains across patient-physical therapist perspectives.

ICF category	Description	Patient's perspective		Physical therapist's perspective	
		Mean (SD)	Median [IQR]	Mean (SD)	Median [IQR]
b235	Vestibular functions	17.1 (25.9)	0.0 [0.0, 30.0]	13.4 (22.3)	0.0 [0.0, 21.0]
b240	Sensations associated with hearing and vestibular function	12.0 (22.7)	0.0 [0.0, 13.0]	10.2 (20.5)	0.0 [0.0, 10.0]
b260	Proprioceptive function	17.3 (26.1)	0.0 [0.0, 30.0]	16.1 (23.2)	0.0 [0.0, 28.5]
b265	Touch function	15.7 (25.9)	0.0 [0.0, 21.5]	12.0 (21.5)	0.0 [0.0, 16.5]
b270	Sensory functions related to temperature and other stimuli	11.9 (23.3)	0.0 [0.0, 11.0]	10.6 (21.0)	0.0 [0.0, 12.5]
b280	Sensation of pain	55.7 (29.0)	60.0 [35.0, 80.0]	50.8 (26.4)	51.0 [30.0, 71.0]
b455	Exercise tolerance functions	37.6 (31.8)	37.0 [4.0, 64.5]	35.9 (29.6)	35.0 [4.0, 60.0]
b710	Mobility of joint functions	41.7 (31.6)	40.0 [10.0, 70.0]	33.8 (27.1)	30.0 [8.5, 54.0]
b715	Stability of joint functions	21.4 (29.4)	3.0 [0.0, 39.0]	15.5 (23.8)	0.0 [0.0, 24.0]
b720	Mobility of bone functions	25.2 (29.0)	12.0 [0.0, 48.0]	21.9 (25.5)	11.0 [0.0, 40.0]
b730	Muscle power functions	34.5 (30.9)	30.0 [0.0, 60.0]	30.2 (27.3)	26.0 [0.0, 50.0]
b735	Muscle tone functions	27.3 (29.7)	19.0 [0.0, 50.0]	23.7 (24.9)	17.0 [0.0, 41.5]
b740	Muscle endurance functions	33.4 (31.4)	29.0 [0.0, 62.0]	31.0 (28.5)	25.0 [0.0, 50.0]
b750	Motor reflex functions	10.3 (21.5)	0.0 [0.0, 8.0]	7.7 (17.0)	0.0 [0.0, 2.0]
b760	Control of voluntary movement functions	13.5 (24.0)	0.0 [0.0, 19.8]	11.2 (19.4)	0.0 [0.0, 16.8]
b770	Gait pattern functions	24.0 (30.4)	5.0 [0.0, 48.0]	20.6 (27.1)	4.0 [0.0, 39.0]
b780	Sensations related to muscles and movement functions	39.0 (29.7)	39.0 [9.0, 62.5]	34.4 (25.8)	35.0 [10.0, 50.0]
d410	Changing basic body position	29.4 (30.4)	20.0 [0.0, 50.5]	25.3 (27.3)	17.0 [0.0, 48.5]
d415	Maintaining a body position	27.2 (30.3)	15.0 [0.0, 50.0]	22.7 (27.0)	10.0 [0.0, 40.0]
d420	Transferring oneself	18.1 (25.8)	1.0 [0.0, 32.0]	15.7 (23.6)	0.0 [0.0, 25.0]
d430	Lifting and carrying objects	27.3 (30.2)	15.0 [0.0, 50.0]	24.7 (28.2)	11.0 [0.0, 47.5]
d440	Fine hand use	11.5 (23.3)	0.0 [0.0, 8.8]	9.3 (20.2)	0.0 [0.0, 5.0]
d445	Hand and arm use	17.4 (26.4)	0.0 [0.0, 30.0]	15.5 (23.9)	0.0 [0.0, 25.0]
d450	Walking	23.6 (30.0)	5.0 [0.0, 45.0]	21.7 (27.1)	6.0 [0.0, 40.0]
d540	Dressing	14.9 (23.3)	0.0 [0.0, 23.5]	13.6 (20.8)	0.0 [0.0, 20.0]
d570	Looking after one's health	7.2 (17.0)	0.0 [0.0, 3.0]	7.7 (16.5)	0.0 [0.0, 4.0]
d999	Community, social and civic life, unspecified	15.6 (25.2)	0.0 [0.0, 25.0]	16.1 (25.0)	0.0 [0.0, 27.0]
e110	Products or substances for personal consumption	34.0 (34.3)	27.0 [0.0, 59.0]	33.5 (32.7)	30.0 [0.0, 52.8]
e120	Products and technology for personal indoor and outdoor mobility and transportation	25.4 (35.0)	0.0 [0.0, 50.0]	25.4 (34.7)	0.0 [0.0, 50.0]
e310	Immediate family	43.3 (38.5)	45.0 [0.0, 80.0]	44.2 (38.0)	50.0 [0.0, 80.0]
e320	Friends	38.3 (38.5)	30.0 [0.0, 75.0]	39.0 (38.0)	34.5 [0.0, 75.0]
e340	Personal care providers and personal assistants	18.5 (32.5)	0.0 [0.0, 25.0]	18.2 (32.3)	0.0 [0.0, 21.0]
e355	Health professionals	55.5 (37.6)	63.0 [13.5, 90.0]	54.7 (35.0)	58.0 [24.2, 85.0]
e580	Health services, systems and policies	50.5 (34.0)	50.0 [19.2, 80.0]	48.0 (31.2)	50.0 [25.2, 70.8]

ICF, classification of functioning, disability and health; IQR, interquartile range (Q1, Q3); SD, standard deviation; VAS, visual analog scale.

Patient ratings reflect patient-perceived relevance, whereas physical therapist ratings reflect professional assessment. Environmental factor ratings reflect contextual relevance without distinguishing whether the factor acted as a barrier or facilitator.

**Table 4:** Magnitude, correspondence, and individual-level differences between patient-reported and physical therapist-assessed VAS ratings across ICF categories.

ICF category	Description	Valid dyads, n	Mean paired difference, mm (95% CI)	Wilcoxon signed-rank test (*Z*)	Unadjusted *p*-value (2-tailed)	Holm-adjusted *p*-value	Effect side *r*	ICC(2,1) (95%CI)	Absolute difference >15 mm, *n* (%)
b235	Vestibular functions	387	3.78 (2.55 to 5.14)	−6.08	<0.001	<0.001	0.309	0.84 (0.76 to 0.91)	37 (9.6%)
b240	Sensations associated with hearing and vestibular function	387	1.78 (0.72 to 2.94)	−4.19	<0.001	<0.001	0.213	0.86 (0.77 to 0.94)	22 (5.7%)
b260	Proprioceptive function	386	1.16 (0.01 to 2.35)	−1.51	0.131	0.920	0.077	0.89 (0.85 to 0.92)	47 (12.2%)
b265	Touch function	386	3.68 (2.33 to 5.13)	−5.54	<0.001	<0.001	0.282	0.82 (0.74 to 0.89)	51 (13.2%)
b270	Sensory functions related to temperature and other stimuli	387	1.37 (0.60 to 2.19)	−3.75	<0.001	0.004	0.191	0.94 (0.90 to 0.96)	25 (6.5%)
b280	Sensation of pain	386	4.77 (3.19 to 6.41)	−6.47	<0.001	<0.001	0.329	0.82 (0.77 to 0.87)	81 (21.0%)
b455	Exercise tolerance functions	386	1.69 (0.25 to 3.17)	−2.12	0.034	0.338	0.108	0.89 (0.85 to 0.92)	68 (17.6%)
b710	Mobility of joint functions	386	7.83 (5.70 to 9.97)	−6.99	<0.001	<0.001	0.356	0.70 (0.62 to 0.76)	113 (29.3%)
b715	Stability of joint functions	387	5.91 (4.14 to 7.74)	−6.50	<0.001	<0.001	0.330	0.76 (0.67 to 0.83)	66 (17.1%)
b720	Mobility of bone functions	387	3.29 (1.58 to 5.19)	−3.66	<0.001	0.005	0.186	0.78 (0.70 to 0.84)	80 (20.7%)
b730	Muscle power functions	387	4.29 (2.39 to 6.26)	−3.50	<0.001	0.008	0.178	0.77 (0.70 to 0.83)	88 (22.7%)
b735	Muscle tone functions	387	3.65 (1.92 to 5.44)	−3.75	<0.001	0.004	0.191	0.78 (0.71 to 0.84)	84 (21.7%)
b740	Muscle endurance functions	387	3.02 (1.56 to 4.52)	−3.59	<0.001	0.006	0.182	0.87 (0.82 to 0.91)	71 (18.3%)
b750	Motor reflex functions	387	2.59 (1.17 to 4.10)	−3.76	<0.001	0.004	0.191	0.70 (0.57 to 0.81)	45 (11.6%)
b760	Control of voluntary movement functions	386	2.32 (0.87 to 3.90)	−2.12	0.034	0.338	0.108	0.75 (0.65 to 0.84)	48 (12.4%)
b770	Gait pattern functions	385	3.44 (1.91 to 5.01)	−3.91	<0.001	0.002	0.199	0.85 (0.81 to 0.89)	57 (14.8%)
b780	Sensations related to muscles and movement functions	387	4.55 (3.10 to 6.05)	−6.02	<0.001	<0.001	0.306	0.85 (0.81 to 0.89)	69 (17.8%)
d410	Changing basic body position	387	4.07 (2.75 to 5.51)	−6.17	<0.001	<0.001	0.314	0.88 (0.83 to 0.92)	57 (14.7%)
d415	Maintaining a body position	387	4.55 (3.15 to 6.03)	−6.18	<0.001	<0.001	0.314	0.86 (0.81 to 0.91)	54 (14.0%)
d420	Transferring oneself	387	2.42 (1.23 to 3.73)	−3.64	<0.001	0.005	0.185	0.87 (0.81 to 0.92)	45 (11.6%)
d430	Lifting and carrying objects	387	2.68 (1.36 to 4.07)	−3.39	<0.001	0.011	0.172	0.89 (0.84 to 0.93)	40 (10.3%)
d440	Fine hand use	385	2.25 (1.18 to 3.41)	−4.02	<0.001	0.001	0.205	0.87 (0.79 to 0.93)	32 (8.3%)
d445	Hand and arm use	384	1.92 (0.90 to 3.04)	−3.18	0.001	0.022	0.162	0.91 (0.86 to 0.94)	37 (9.6%)
d450	Walking	385	1.85 (0.43 to 3.26)	−2.55	0.011	0.118	0.130	0.87 (0.83 to 0.91)	58 (15.1%)
d540	Dressing	387	1.31 (0.32 to 2.40)	−2.05	0.041	0.338	0.104	0.89 (0.82 to 0.94)	31 (8.0%)
d570	Looking after one's health	387	−0.56 (−1.52 to 0.40)	−1.36	0.175	1.000	0.069	0.84 (0.76 to 0.90)	25 (6.5%)
d999	Community, social and civic life, unspecified	387	−0.55 (−1.53 to 0.39)	−0.24	0.814	1.000	0.012	0.93 (0.89 to 0.95)	28 (7.2%)
e110	Products or substances for personal consumption	386	0.46 (−0.97 to 1.94)	−0.30	0.768	1.000	0.015	0.91 (0.86 to 0.94)	46 (11.9%)
e120	Products and technology for personal indoor and outdoor mobility and transportation	384	−0.17 (−1.16 to 0.85)	−0.73	0.468	1.000	0.037	0.96 (0.94 to 0.98)	32 (8.3%)
e310	Immediate family	385	−0.94 (−2.16 to 0.34)	−2.67	0.008	0.099	0.136	0.95 (0.91 to 0.97)	24 (6.2%)
e320	Friends	384	−0.63 (−1.69 to 0.51)	−2.58	0.010	0.118	0.132	0.96 (0.93 to 0.98)	23 (6.0%)
e340	Personal care providers and personal assistants	369	0.22 (−0.71 to 1.25)	−0.25	0.802	1.000	0.013	0.96 (0.92 to 0.98)	22 (6.0%)
e355	Health professionals	386	0.81 (−0.69 to 2.27)	−0.90	0.368	1.000	0.046	0.92 (0.89 to 0.94)	54 (14.0%)
e580	Health services, systems and policies	385	2.29 (0.57 to 4.05)	−2.84	0.005	0.063	0.145	0.86 (0.80 to 0.90)	61 (15.8%)

ICC, intraclass correlation coefficient; ICF, classification of functioning, disability and health.

Paired differences were calculated as patient rating minus physical therapist rating; positive values indicate higher patient ratings and negative values indicate higher physical therapist ratings. The sign of the Wilcoxon *Z* statistic was not used to determine direction. Effect size *r* was calculated as |*Z*|/√*N*. Holm-adjusted *p*-values account for the 34 paired comparisons. ICC values represent two-way random-effects, absolute-agreement, single-measure coefficients (ICC[2,1]) and are interpreted descriptively. The 15-mm value is an interpretive benchmark and not an MCID or a validated cutoff for patient-physical therapist disagreement.

Within the Body Functions (b) component, absolute paired-score correspondence ranged from moderate to excellent (ICC[2,1] range: 0.697–0.935). Statistically significant differences were observed in 16 of the 17 categories using unadjusted *p*-values, of which 14 remained significant after Holm adjustment. Patients generally reported slightly higher VAS scores than physical therapists. The largest mean difference was documented for category “b710 Mobility of joint functions” (*Z* = −6.988, unadjusted *p* < 0.001, Holm-adjusted *p* < 0.001), where patients ratings were, on average, 7.83 mm (95% CI: 5.70–9.97; *r* = 0.356) higher than physical therapist ratings. This was followed by “b715 Stability of joint functions” (*Z* = −6.495, unadjusted and Holm-adjusted *p* < 0.001; mean difference = 5.91 mm, 95% CI: 4.14–7.74 mm; *r* = 0.330) and “b280 Sensation of pain” (*Z* = −6.470, unadjusted and Holm-adjusted *p* < 0.001; mean difference = 4.77 mm, 95% CI: 3.19–6.41 mm; *r* = 0.329). Conversely, no statistically significant difference was detected for category “b260 Proprioceptive function” (*Z* = −1.509, unadjusted *p* = 0.131; Holm-adjusted *p* = 0.920). Differences for b455 Exercise tolerance functions and b760 Control of voluntary movement functions were significant before adjustment but did not remain significant after the Holm procedure. Because all absolute mean differences within this component remained below the 15-mm benchmark, the differences were considered limited in magnitude at the group level, although individual-level discrepancies were observed in some categories. In particular, 113 of the 386 valid dyads (29.3%) differed by more than 15 mm for “b710 Mobility of joint functions”.

Regarding the Activities and Participation (d) component, absolute paired-score correspondence ranged from good to excellent (ICC[2,1] range: 0.835–0.926), while the group-level mean differences were generally small. Statistically significant differences were identified in 8 of the 10 categories using unadjusted *p*-values, of which 6 remained significant after Holm adjustment. Patients generally reported higher VAS scores than physical therapists in the categories showing positive mean differences. The largest differences within this component were found in “d415 Maintaining a body position” (*Z* = −6.175, unadjusted and Holm-adjusted *p* < 0.001; mean difference = 4.55 mm, 95% CI: 3.15–6.03 mm; *r* = 0.314) and “d410 Changing basic body position” (*Z* = −6.174, unadjusted and Holm-adjusted *p* < 0.001; mean difference = 4.07 mm, 95% CI: 2.75–5.51 mm; *r* = 0.314). In contrast, no statistically significant differences were observed for “d570 Looking after one's health” (*Z* = −1.357, unadjusted *p* = 0.175; Holm-adjusted *p* = 1.000) or “d999 Community, social and civic life, unspecified” (*Z* = −0.235, unadjusted *p* = 0.814; Holm-adjusted *p* = 1.000). The differences observed for “d450 Walking” and d540 Dressing did not remain significant after adjustment for multiple comparisons. None of the mean differences within this component reached the 15-mm benchmark.

Finally, the Environmental Factors (e) component demonstrated good to excellent absolute paired-score correspondence, with ICC values ranging from 0.855 to 0.958. Mean differences were small and varied in direction. No statistically significant differences were observed for physical context categories, including e110 (unadjusted *p* = 0.768; Holm-adjusted *p* = 1.000) and e120 (unadjusted *p* = 0.468; Holm-adjusted *p* = 1.000), nor for formal support mechanisms like e340 (unadjusted *p* = 0.802; Holm-adjusted *p* = 1.000) and e355 (unadjusted *p* = 0.368; Holm-adjusted *p* = 1.000). Before adjustment, statistically significant differences were observed for “e580 Health services, systems, and policies” (*Z* = −2.840, unadjusted *p* = 0.005; mean difference = 2.29 mm; *r* = 0.145), for which patients reported higher ratings, as well as “e310 Immediate family” (*Z* = −2.668, unadjusted *p* = 0.008; mean difference = −0.94 mm; *r* = 0.136) and “e320 Friends” (*Z* = −2.581, unadjusted *p* = 0.010; mean difference = −0.63 mm; *r* = 0.132), for which physical therapists reported slightly higher ratings than patients. However, none of these differences remained statistically significant after Holm adjustment, with adjusted *p*-values of 0.063, 0.099, and 0.118, respectively. None of the mean differences within the Environmental Factors component approached the 15-mm benchmark. These results refer to correspondence and differences in the degree to which Environmental Factors were identified as relevant to functioning; they do not indicate whether the factors acted as barriers or facilitators.

Across the 34 ICF categories, the proportion of individual dyads with an absolute patient-physical therapist difference greater than 15 mm ranged from 5.7% to 29.3%, with a median of 12.3%. Thus, small group-level mean differences and generally substantial paired-score correspondence coexisted with potentially informative discrepancies in some individual dyads (see Table 4).

## 4 Discussion

The findings of this study provide empirical support for the prevalence and the perceived relevance of the categories included in the specific ICF Core Set for the management of musculoskeletal conditions in primary care physical therapy services in Spain. All 34 categories exceeded the predefined empirical retention threshold, and their retention remained stable across broad levels of disability and comorbidity burden. The study also provides a novel examination of the correspondence and differences between patient-reported and physical therapist-assessed perspectives in real-world clinical practice. Rather than representing interchangeable assessments, these perspectives provide complementary information about the lived experience and professional assessment of functioning. Together, these findings establish a preliminary empirical framework for identifying the functioning domains that may support systematic documentation in this clinical setting. The prevalence of functioning-related problems or frequency of contextual relevance across all 34 evaluated ICF categories exceeded the predefined 5% retention threshold. Retention criteria vary across cross-sectional studies, generally ranging from 5% to 20%, although higher thresholds have occasionally been applied (40). Although this variability limits direct comparisons between studies, the lowest-prevalence category in the overall sample (d570 Looking after one's health) reached 23.3% and therefore remained above the thresholds most commonly reported in the literature. Nevertheless, these findings should be interpreted as prevalence-based empirical support for category retention rather than as a complete psychometric evaluation of content validity.

The sensitivity analyses further supported the stability of the category-retention findings. All 34 ICF categories remained above the predefined 5% threshold in the subgroups defined by the observed medians of WHODAS 2.0 and SCQ. Because dichotomization may reduce information from continuous variables, these subgroup analyses were interpreted only as pragmatic assessments of retention stability. Complementary analyses preserving WHODAS 2.0 and SCQ as continuous variables provided additional information. Higher WHODAS 2.0 scores showed moderate positive associations with patient-reported ratings for Body Functions (b) and Activities and Participation (d), whereas SCQ scores showed weaker positive associations with the same functioning components. These findings suggest that patients with greater disability, and to a lesser extent, greater comorbidity burden tended to report higher overall ratings of functioning-related problems. This pattern is consistent with previous evidence showing that multimorbidity is associated with a greater burden of disability and functional limitations (41). However, the correlations were not strong, indicating that WHODAS 2.0, comorbidity burden, and the ICF category ratings capture related but non-identical aspects of functioning. Neither WHODAS 2.0 nor SCQ was significantly associated with the Environmental Factors summary rating. This finding should not be interpreted as evidence that environmental context is unrelated to disability or comorbidity, because the ICF conceptualizes functioning and disability as outcomes of the dynamic interaction between health conditions and contextual factors, including environmental barriers and facilitators (13). In the present study, Environmental Factor ratings were intended to identify contextual relevance rather than the direction of influence. Consequently, averaging factors that could operate in opposing directions may have limited the interpretability of their associations with disability and comorbidity burden. Future studies should assess barriers and facilitators separately when examining the relationship between environmental context and functioning.

The empirical relevance of the ICF Core Set categories was supported by the correspondence between the frequency with which the categories were identified and the magnitude of their VAS ratings. Sensation of pain (b280) emerged as the most prominent category, exhibiting both the highest prevalence (>90%) and one of the highest VAS rating. A high-prevalence clinical cluster was also observed within the 60–90% range, in which “b710 Mobility of joint functions” was the most prevalent Body Functions category, and “d430 Lifting and carrying objects” was the most frequently identified activity limitation. These findings are consistent with the close interrelationship between pain, movement impairments, and disability in musculoskeletal conditions (42). Furthermore, movement dysfunction is closely intertwined with psychological risk factors, such as fear-avoidance, catastrophizing, and depression (43). Although persistent pain may alter muscle activation and movement patterns, structured and graded movement may be used therapeutically to modulate pain and support functional recovery (44). Such strategies have already been successfully implemented within primary care settings (45).

Regarding Environmental Factors (e), the findings showed a high frequency of patient-reported contextual relevance for institutional support structures, with health services (e580, 80.6%) and health professionals (e355, 79.8%) emerging as the most prominent categories. The substantial relevance attributed to these categories aligns with international key quality indicators for primary care musculoskeletal services, which emphasize the need to resource, manage, and monitor healthcare systems and provider availability to optimize clinical pathways (46). This contextual relevance may be particularly important in Castilla y León (Spain), a geographically extensive region characterized by population dispersion and the organization of primary care services across urban and rural areas. Previous evidence has shown that healthcare accessibility, geographical deprivation, and characteristics of the primary care setting may influence healthcare referral pathways (47). However, because the present study did not compare Environmental Factor ratings according to rural or urban residence, these findings cannot establish geographical inequalities in access to care. Finally, social relationships also proved highly relevant for patients, represented by immediate family (e310, 67.5%) and friends (e320, 60.8%), with a moderate contextual influence. These findings are consistent with those obtained in the clinical validation of the ICF Core Set for post-acute musculoskeletal conditions in this setting (21). Altogether, these findings illustrate the inherent complexity of musculoskeletal conditions and highlight the critical need to adopt a biopsychosocial approach for their management (48), further underscoring the potential clinical utility of an ICF Core Set for systematically documenting these functioning domains in routine physical therapy. The multicenter design, relatively large sample, inclusion of both patient and physical therapist perspectives, and use of complementary analyses of magnitude, correspondence, and individual-level differences represent important strengths of the study.

The definition of ICF domains represents a key stage in developing standardized assessment and reporting systems (StARS). As described by Stucki et al. (24), this process entails: (a) determining meaningful functioning elements to be measured, (b) deciding the perspective to be adopted, (c) selecting appropriate measurement instruments, and (d) defining the reporting approach, with an increasing emphasis on interval-based metrics (49). While certain ICF Core Sets can provide a preliminary approach to document functioning (e.g., Generic Set or Rehabilitation Set), relying on a tailored instrument ensures that clinical assessments will be focused on functioning domains that have proven most significant for a specific clinical setting (15, 25). When compared to the Rehabilitation Set, the specific ICF Core Set empirically evaluated in the present study shares essential ICF categories regarding pain (b280), neuromusculoskeletal functions (e.g., b710 Mobility of joint functions), and mobility (e.g., d450 Walking). However, the specific ICF Core Set includes additional critical categories for describing human movement, encompassing neuromotor control and sensorimotor functions. Conversely, it eliminates external categories less relevant to physical therapists within this musculoskeletal scope, such as metabolic or genitourinary functions. Furthermore, a small group of environmental factors is included to capture the primary care context, a dimension that is lacking in the Rehabilitation Set (18, 22).

Since the primary care context is inherently multidisciplinary, there is an opportunity to develop a specific Clinical Assessment Schedule (CLAS) that integrates the recording of functioning data through the active participation of all healthcare professionals. This approach aligns with the international frameworks developed by the European Union of Medical Specialists Physical and Rehabilitation Medicine (UEMS-PRM) Section and Board, which aim to standardize the documentation of functioning in rehabilitation (50). Based on the ICF framework, these schedules define which aspects to assess and which tools to utilize according to the specific service type, with the ultimate goal of harmonizing professional standards, ensuring equity, and enabling the comparison of outcomes. Although this standardization could contribute to the optimization of rehabilitation services (51), the primary care team's limited understanding of the physical therapist's roles could pose a barrier to its development and implementation (52). Consequently, leadership from the physical therapy profession may help facilitate interdisciplinary initiatives, advocate for the systemic integration of the ICF framework, and clarify clinical roles within primary care networks (53). Despite these challenges, ICF-based tools have been successfully developed for different musculoskeletal conditions (54).

Another critical consideration in the development of StARS is the selection of which perspective to adopt when documenting functioning. Within the “Activities and Participation” (d) component, the operational options are linked to the concepts of capacity, reflecting functioning in a standardized environment; performance, reflecting functioning in the person's current environment; and the individual's subjective appraisal of participation and functioning (24). This study provides an exploratory examination of the relationship between patient-reported lived experience and physical therapist-assessed clinical relevance in primary care.

Absolute paired-score correspondence ranged from moderate to excellent across the 34 ICF categories. However, the ICCs were interpreted descriptively and should not be considered evidence of strict inter-rater reliability or interchangeability between patients and physical therapists. Patient ratings reflect lived experience and perceived relevance, whereas physical therapist ratings reflect professional clinical judgment. Consequently, correspondence indicates similarity between two complementary perspectives, rather than equivalence between two interchangeable measurements. Using unadjusted *p*-values, 27 of the 34 patient-physical therapist comparisons were statistically significant, and 20 remained significant after Holm adjustment. Nevertheless, all group-level mean differences were below the predefined 15-mm interpretive benchmark (30, 55). The largest mean difference was observed for b710 Mobility of joint functions, with patients rating this category 7.83 mm higher than physical therapists on average. Absolute effect sizes ranged from 0.012 to 0.356, with a median of 0.180, indicating that the observed group-level differences were generally small, although moderate effects occurred in a limited number of categories. These results illustrate how a relatively large sample can identify small systematic differences that may be statistically detectable without necessarily being substantial in magnitude. Small group-level differences did not, however, imply complete correspondence within individual dyads. Depending on the ICF category, between 5.7% and 29.3% of patient-physical therapist pairs showed an absolute difference greater than 15 mm, with a median of 12.3% across categories. The highest proportion was observed for b710 Mobility of joint functions, for which 113 dyads (29.3%) exceeded this benchmark. These findings demonstrate that overall paired-score correspondence can coexist with potentially informative individual-level differences. Such differences should not necessarily be interpreted as measurement error or poor reliability. Instead, they may identify functioning domains in which the patient's lived experience and the physical therapist's professional assessment differ. Clinicians should therefore remain attentive to individual patient-professional discrepancies and use them as opportunities for further communication, clarification of priorities, and shared assessment. Addressing these differences through a strong therapeutic alliance (56) may support person-centered care and shared decision-making throughout the rehabilitation pathway (57). The findings of this study provide an empirical foundation for the future development of a StARS for primary care physical therapy services in Spain. Such a system could subsequently be evaluated across different regions to determine its applicability within the Spanish National Health System. In addition to deciding which areas of functioning to document and which perspective to adopt, it is necessary to choose tools based on or linked to the ICF. These tools should enable the collection and recording of functioning data in order to develop comprehensive, ICF-based systems (54, 58, 59). Some authors have already highlighted the advantages of the interval scale metric in the literature because it is well-suited to the dynamic and continuous nature of functioning. Additionally, this reporting approach allows for the documentation of clinical changes and comparison of functional status among patients and populations (49, 60).

An alternative approach to operationalizing an ICF Core Set is illustrated by the Clinical Functioning Information Tool (ClinFIT) (61). ClinFIT is an ICF-based tool derived from the ICF Core Set Generic-30 (Rehabilitation Set) and supported by psychometric development and validation studies (62–64). ClinFIT has been usedto evaluate functioning and detect changes over time and across different health conditions (65), and its extension to primary care has also been proposed (66). The musculoskeletal ClinFIT version includes 17 ICF categories (67), 12 of which, representing approximately 70%, overlap with the specific ICF Core Set examined in the present study. This overlap suggests that both approaches capture foundational musculoskeletal functioning domains, while the present Core Set includes additional sensorimotor, movement-related, and contextual categories relevant to primary care physical therapy.

The implementation of such ICF-based tools highlights the potential transferability of the present findings. Although the study was conducted within a regional healthcare service, the results may be relevant to other settings within the Spanish National Health System that have comparable primary care physical therapy structures. However, transferability should be evaluated empirically rather than assumed, particularly across countries with different healthcare organizations, referral pathways, professional roles, and access to physical therapy services. Further multicenter and international research is therefore needed to examine whether the same category-retention patterns and patient-professional relationships are observed in other health systems.

### 4.1 Limitations

This study has several limitations that should be considered when interpreting the findings. Overall, the cross-sectional design represents an overarching constraint, as it prevents the assessment of longitudinal measurement properties. Consequently, responsiveness to clinical change, predictive validity, and test-retest reliability could not be evaluated, warranting future prospective investigation.

Regarding the primary objective of empirically evaluating category retention, several limitations arose from the recruitment strategy and structural design. Although recruitment was conducted across multiple primary care centers, all participants were recruited from a single public healthcare provider (SACYL) in Spain using a non-probability sampling strategy. The sample should therefore not be considered representative of the general population with musculoskeletal conditions, and transferability to private services, other Spanish regions, or international healthcare systems may be limited. Recruitment of patients attending public physical therapy services may also have favored individuals with greater disability or healthcare needs. Although the sensitivity analyses showed stable category retention across broad levels of disability and comorbidity burden, they cannot eliminate potential selection bias. The predefined 5% threshold was used as an empirical category-retention criterion and should not be interpreted as a complete psychometric assessment of content validity. In addition, the exclusion of Body Structures (s) and Personal Factors limited the assessment of anatomical impairments and individual modifiers of functioning. Environmental Factor ratings were intended to identify contextual relevance rather than the direction of influence. Consequently, the estimates represent frequencies of contextual relevance and do not indicate whether the factors acted as barriers or facilitators.

Regarding the secondary objective of examining correspondence and differences between patient-reported and physical therapist-assessed perspectives, several analytical and conceptual limitations should be acknowledged. Patient and physical therapist ratings reflect complementary rather than interchangeable perspectives. Therefore, ICC values should be interpreted descriptively as indicators of absolute paired-score correspondence and not as evidence of conventional inter-rater reliability. The large sample size also increased the likelihood of detecting statistically significant differences of limited magnitude. Although interpretation was supported by Holm-adjusted *p*-values, confidence intervals, effect sizes, and individual-level absolute differences, the clinical implications of these discrepancies remain uncertain. Finally, the 15 mm value was used only as a conservative descriptive benchmark and has not been validated as a cutoff for cross-sectional patient-physical therapist disagreement. Similarly, the component summary ratings used in the continuous sensitivity analyses were study-specific analytical constructs rather than independently validated scales. The corresponding associations with WHODAS 2.0 and SCQ should therefore be regarded as exploratory and not as evidence of causality or formal convergent validity.

### 4.2 Conclusions

This multicenter study provides empirical evidence regarding the prevalence and perceived relevance of the categories included in the specific ICF Core Set for the management of musculoskeletal conditions in primary care physical therapy services in Spain. All evaluated categories exceeded the predefined empirical retention threshold, and their retention remained stable across broad levels of disability and comorbidity burden. The study also provides information on the correspondence and differences between patient-reported and physical therapist-assessed perspectives on functioning. Although substantial paired-score correspondence was observed at the group level, individual differences between these complementary perspectives may provide clinically informative insights for person-centered assessment and shared decision-making. These findings support further evaluation of the specific ICF Core Set as a framework for standardized documentation of functioning in primary care physical therapy. Future research should examine its feasibility, measurement properties, responsiveness, implementation processes, and impact on clinical decision-making and patient-centered care.

## Data Availability

The raw data supporting the conclusions of this article will be made available by the authors, without undue reservation.
