# Supplementary material for: Empirical evaluation of an ICF core set for managing musculoskeletal conditions in primary care physical therapy services in Spain: a multicenter study
Source: Front Rehabil Sci. 2026 Sep 22;7:1960608. doi: 10.3389/fresc.2026.1960608 (PMC13638589; doi:10.3389/fresc.2026.1960608)
Supplement: Supplementary file 1 [file Datasheet1.pdf]

Table S1. Pragmatic median-split sensitivity analysis of the 5% retention threshold

Variable	Observed median	Group	Group n	Categories exceeding 5%	Minimum prevalence, %	Maximum prevalence, %
WHODAS 2.0	16.7	≤ median (16.7)	184	34/34	16.3	91.8
WHODAS 2.0	16.7	> median (16.7)	180	34/34	30.0	96.1
SCQ	4.0	≤ median (4.0)	192	34/34	20.3	93.8
SCQ	4.0	> median (4.0)	191	34/34	26.7	94.8

Abbreviations: SCQ: Self-Administered Comorbidities Questionnaire; WHODAS 2.0: World Health Organization Disability Assessment Schedule 2.0

Table S2. Sensitivity analyses preserving WHODAS 2.0 and SCQ as continuous variables

Variable	ICF summary rating	Valid n	Spearman ρ	95% CI	Unadjusted p	Holm-adjusted p
WHODAS 2.0 total score	Body Functions (b) mean VAS	364	0.351	0.253 to 0.447	<0.001	<0.001
WHODAS 2.0 total score	Activities and Participation (d) mean VAS	364	0.329	0.226 to 0.430	<0.001	<0.001
WHODAS 2.0 total score	Functioning categories mean VAS (b+d)	364	0.357	0.257 to 0.452	<0.001	<0.001
WHODAS 2.0 total score	Environmental Factors (e) mean VAS	361	0.101	0.000 to 0.200	0.055	0.055
SCQ total score	Body Functions (b) mean VAS	383	0.197	0.098 to 0.298	<0.001	<0.001
SCQ total score	Activities and Participation (d) mean VAS	383	0.178	0.075 to 0.275	<0.001	<0.001
SCQ total score	Functioning categories mean VAS (b+d)	383	0.202	0.104 to 0.299	<0.001	<0.001
SCQ total score	Environmental Factors (e) mean VAS	380	0.054	-0.051 to 0.157	0.289	0.289

Abbreviations: CI: Confidence Interval; ICF: International Classification of Functioning, Disability and Health; SCQ: Self-Administered Comorbidities Questionnaire; VAS: Visual Analog Scale; WHODAS 2.0: World Health Organization Disability Assessment Schedule 2.0

This is a provisional file, not the final typeset article
